# Red Plaque on the Dorsal Hand With Necrosis: Think Before You Amputate

**DOI:** 10.5826/dpc.1004a100

**Published:** 2020-10-26

**Authors:** Marc Mir-Bonafé, Javier Aubán-Pariente, Sheila Requena-López, Celia Gómez-de-Castro, Yolanda Hidalgo-García

**Affiliations:** 1 Department of Dermatology, Hospital Universitario Central de Asturias, Oviedo, Spain

**Keywords:** cellulitis, neutrophilic dermatosis, neutrophilic dermatosis of the dorsal hands, Sweet syndrome

## Case Presentation

**Figure 1:**
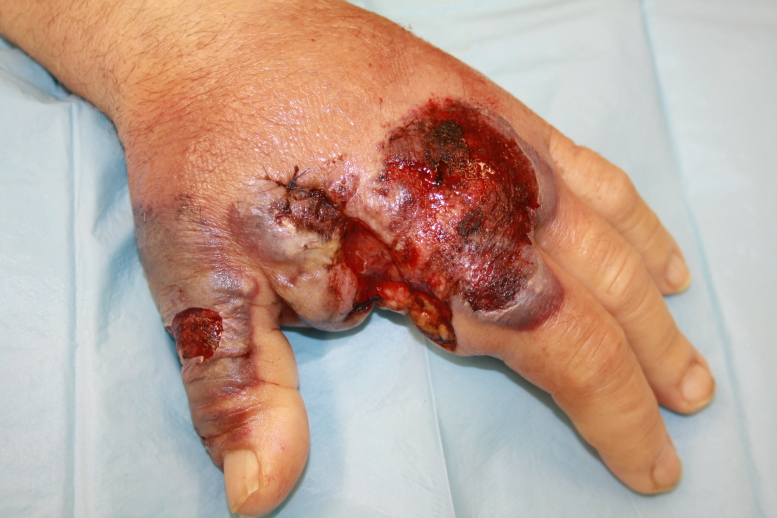
Ulcers with violaceous borders and surrounding edema present on the dorsal left hand and aspects of the thumb with proximal metacarpophalangeal joints of the third and fourth fingers. Note the absence of the left index finger.

An 82-year-old man presented with fever and an erythematoedematous left index finger. He had a history of myelodysplastic syndrome. He was placed on intravenous antibiotic (amoxicillin/clavulanic acid) due to suspicion of cellulitis. The condition gradually developed blisters and necrosis, and surgeons decided to amputate. In the following days, the clinical picture worsened with the development of ulcers with violaceous borders and surrounding edema on the dorsal hand and proximal metacarpophalangeal joints of the thumb and third and fourth fingers (Figure 1). Skin biopsy was compatible with a neutrophilic dermatosis, and the diagnosis of neutrophilic dermatosis of the dorsal hands (NDDH) was made. He was immediately started on oral prednisone 80 mg per day and showed rapid improvement.

## Teaching Point

NDDH, a distributional variant of Sweet syndrome, is an under-recognized entity. It is often clinically misdiagnosed as an infectious process. Physicians should suspect this diagnosis in order to avoid unnecessary and damaging procedures.

